# Dataset of Passerine bird communities in a Mediterranean high mountain (Sierra Nevada, Spain)

**DOI:** 10.3897/zookeys.552.6934

**Published:** 2016-01-13

**Authors:** Antonio Jesús Pérez-Luque, José Miguel Barea-Azcón, Lola Álvarez-Ruiz, Francisco Javier Bonet-García, Regino Zamora

**Affiliations:** 1 Laboratorio de Ecología (iEcolab), Instituto Interuniversitario de Investigación del Sistema Tierra en Andalucía (CEAMA), Universidad de Granada, Avenida del Mediterráneo s/n, 18006, Granada, Spain; 2 Grupo de Ecología Terrestre, Departamento de Ecología, Universidad de Granada, Facultad de Ciencias, Campus de Fuentenueva s/n, 18071, Granada, Spain; 3 Agencia de Medio Ambiente y Agua, Consejería de Medio Ambiente y Ordenación del Territorio (Junta de Andalucía), C/ Joaquina Egüaras 10, E-18013, Granada, Spain

**Keywords:** Passerines, Sierra Nevada (Spain), global-change monitoring, Mediterranean high mountain, species composition, abundance

## Abstract

In this data paper, a dataset of passerine bird communities is described in Sierra Nevada, a Mediterranean high mountain located in southern Spain. The dataset includes occurrence data from bird surveys conducted in four representative ecosystem types of Sierra Nevada from 2008 to 2015. For each visit, bird species numbers as well as distance to the transect line were recorded. A total of 27847 occurrence records were compiled with accompanying measurements on distance to the transect and animal counts. All records are of species in the order Passeriformes. Records of 16 different families and 44 genera were collected. Some of the taxa in the dataset are included in the European Red List. This dataset belongs to the Sierra Nevada Global-Change Observatory (OBSNEV), a long-term research project designed to compile socio-ecological information on the major ecosystem types in order to identify the impacts of global change in this area.

Sierra Nevada Global-Change Observatory

## Introduction

Birds are among the most suitable groups of organisms for assessing species vulnerability to climate change (Pacifi et al. 2015). There is scientific evidence of the impact of climate change on bird communities (Crick 2004, Pearce-Higgins and Green 2014, Pearce-Higgins et al. 2015). Most studies supporting such impacts are based on long-term datasets (e.g. Gregory et al. 2009). Long-term datasets have been recognized as a key component for monitoring biodiversity (Magurran et al. 2010), and are considered one of the major requirements to identify changes in phenology (Sanz 2002). However, long-term monitoring programs are often difficult to develop. In this sense, reviewing old studies can help to integrate short-term studies into long-term datasets, providing a potential source of data to assess changes in ecological communities (Sanz 2002, Müller et al. 2010). This is relevant for the Mediterranean region, where more bird studies as well as available long-term datasets (Sanz 2002) are needed, especially considering that predicted levels in species richness have shown a sharp decrease in the southern regions of Europe (Barbet-Massin et al. 2012).

In this paper, a dataset of passerine bird communities is described from Sierra Nevada, a Mediterranean high mountain region in southern Spain. The dataset comes from Sierra Nevada Global Change Observatory (OBSNEV), a monitoring programme designed to evaluate the potential impacts of global change in this mountain area. Monitoring methodologies of the OBSNEV include revisiting old plots to assess long-term population trends, changes in phenology, and shifts in community composition, among other parameters.

Studies of bird communities in the Sierra Nevada mountain region go back to the 1850s, with the first published records of field observations recorded by ornithologists (Pleguezuelos 1991, Garzón 2012). A recent review of the birds in the Sierra Nevada was made by Garzón and Henares (2012). All these works include passerines, but specific studies focusing specifically on passerine bird communities on this mountain region were conducted during the 1980s (Zamora and Camacho 1984, Zamora 1987a, 1987b, 1988a, 1988b, 1990). The dataset presented here contributes knowledge about the passerines in this area, enabling assessments of population trends (e.g. Zamora and Barea-Azcón 2015).

## Project details

**Project title**: Sierra Nevada Global-Change Observatory (OBSNEV)

**Personnel**: Regino Jesús Zamora Rodríguez (Scientific Coordinator, Principal Investigator, University of Granada); Francisco Javier Sánchez Gutiérrez (Director of the Sierra Nevada National Park and Natural Park).

**Funding**: Sierra Nevada Global Change Observatory is funded by the Consejería de Medio Ambiente y Ordenación del Territorio (Junta de Andalucía) through the European Union (FEDER project) and by the Spanish Government (via "Fundación Biodiversidad", which is a Public Foundation). Some activities undertaken by the OBSNEV (data analysis, quantification of ecosystem services, harmonization of monitoring methods, integration in major cyberinfrastructures, etc.) are funded by the European Commission under different projects (FP7: EU BON; H2020: eLTER, ECOPOTENTIAL; Life: ADAPTAMED).

**Figure 1.**
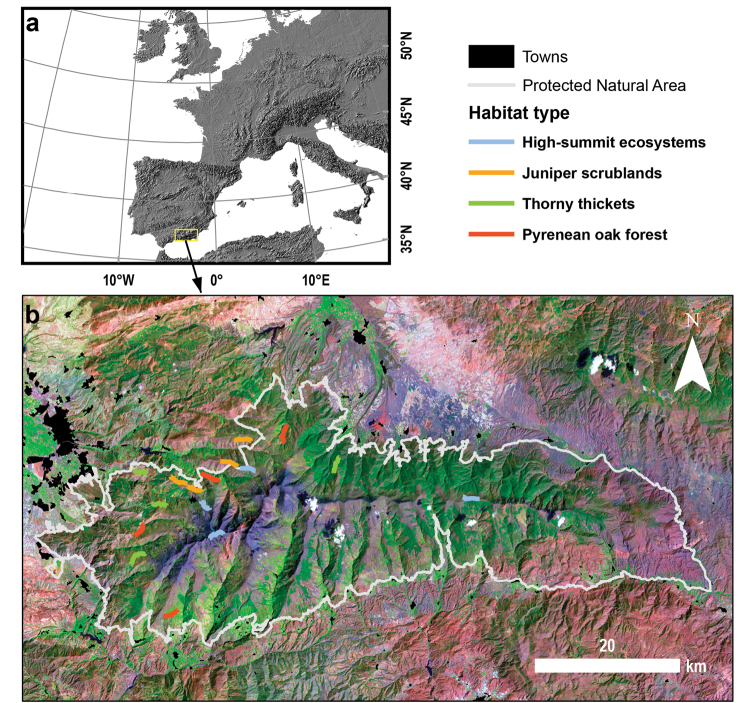
**a** Location of Sierra Nevada (southern Spain) and **b** distribution of transects in the Protected Natural Area of Sierra Nevada. Transect colour according to habitat type (see Methods section). A Landsat 5 Image (2001) was used as background.

**Study area description**: Sierra Nevada (Andalusia, SE Spain), is a mountainous region covering more than 2000 km^2^ with an altitudinal range of between 860 m and 3482 m a.s.l. (Figure 1). The climate is Mediterranean, characterized by cold winters and hot summers, with pronounced summer drought (July-August). The annual average temperature decreases in altitude from 12–16°C below 1500 m to 0°C above 3000 m a.s.l., and the annual average precipitation is about 600 mm. Additionally, the complex orography of the mountains causes sharp climatic contrasts between the sunny, dry south-facing slopes and the shaded, wetter north-facing slopes. Annual precipitation ranges from less than 250 mm in the lowest parts of the mountain range to more than 700 mm in the summit areas. Winter precipitation is mainly in the form of snow above 2000 m a.s.l.

This mountain area harbours 27 habitat types from the Habitat Directive. Sierra Nevada protected area contains at least 78 animal species (48 breeding birds, 17 mammals, 7 invertebrates, 2 amphibians and 4 reptiles) and 13 plant species listed in the Annex II and/or in the Annex IV of Habitat Directive or Annex I or Annex II of Bird Directive. It is thus considered one of the most important biodiversity hotspots in the Mediterranean region (Blanca 1996, Blanca et al. 1998, Cañadas et al. 2014).

Sierra Nevada receives legal protection in multiple ways, including Biosphere Reserve MAB Committee UNESCO; Special Area of conservation (Natura 2000 network); Natural Park and National Park; and IBA (Important Bird Area). The area includes 61 municipalities with more than 90, 000 inhabitants. The main economic activities are agriculture, tourism, livestock raising, beekeeping, mining, and skiing (Bonet et al. 2010).

**Design description**: Sierra Nevada Global Change Observatory (OBSNEV) (Bonet et al. 2011) is a long-term research project that is being undertaken at Sierra Nevada Biosphere Reserve (SE Spain). It is intended to compile the information necessary for identifying as early as possible the impacts of global change, in order to design adequate management mechanisms to minimize these impacts and enable the system to adapt to new environmental conditions (Aspizua et al. 2010, Bonet et al. 2010). The general objectives are to:

Evaluate the functioning of ecosystems in the Sierra Nevada Nature Reserve, their natural processes and dynamics over a medium-term timescale.

Identify population dynamics, phenological changes, and conservation issues regarding key species that could be considered indicators of ecological processes.

Identify the impact of global change on monitored species, ecosystems, and natural resources, providing an overview of trends of change that could help foster ecosystem resilience.

Design mechanisms to assess the effectiveness and efficiency of management activities performed in the Sierra Nevada in order to implement an adaptive management framework.

Help to disseminate information of general interest concerning the values and importance of Sierra Nevada.

The Sierra Nevada Global Change Observatory has four cornerstones:

A monitoring programme with 40 methodologies that collect information on ecosystem functioning (Aspizua et al. 2012, 2014).

An information system to store and manage all the information gathered (http://obsnev.es/linaria.html - Pérez-Pérez et al. 2012; Free access upon registration).

A plan to promote adaptive management of natural resources using the data amassed through the monitoring programme.

An outreach programme to disseminate all the available information to potential users (see News Portal of the project at http://obsnev.es and the wiki of the project at http://wiki.obsnev.es, Pérez-Luque et al. 2012)

The Sierra Nevada Global Change Observatory is linked to other national (Zamora and Bonet 2011) and international monitoring networks: GLOCHAMORE (Global Change in Mountain Regions) (Björnsen 2005), GLOCHAMOST (Global Change in Mountain Sites) (Schaaf 2009), LTER-Spain (Long-Term Ecological Research), LifeWatch (Basset and Los 2012), etc. This project is also involved in several European projects such as MS-MONINA (FP7 project. www.ms-monina.eu), EU BON (Hoffmann et al. 2014), eLTER (H2020 project. www.lter-europe.net/projects/eLTER), ECOPOTENTIAL (H2020 project. www.ecopotential-project.eu/) and ADAPTAMED (Life project).

## Taxonomic coverage

**Figure 2.**
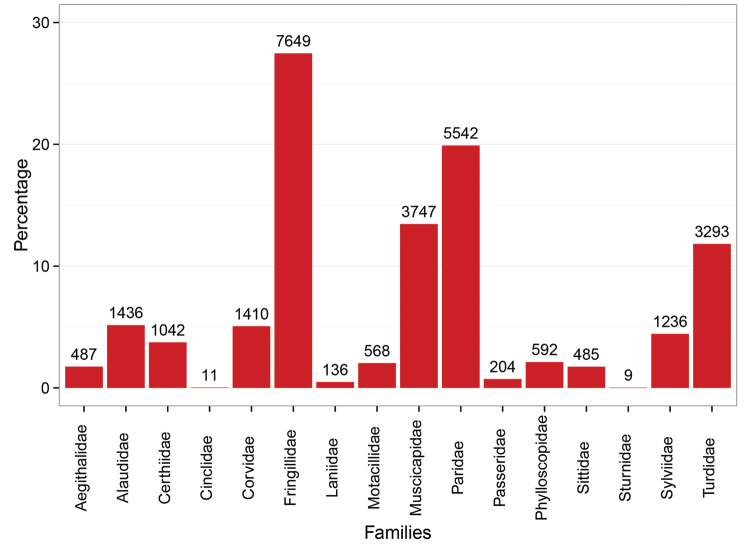
Taxonomic families included in the dataset. The bars show the percentage of records belonging to each family.

**Figure 3.**
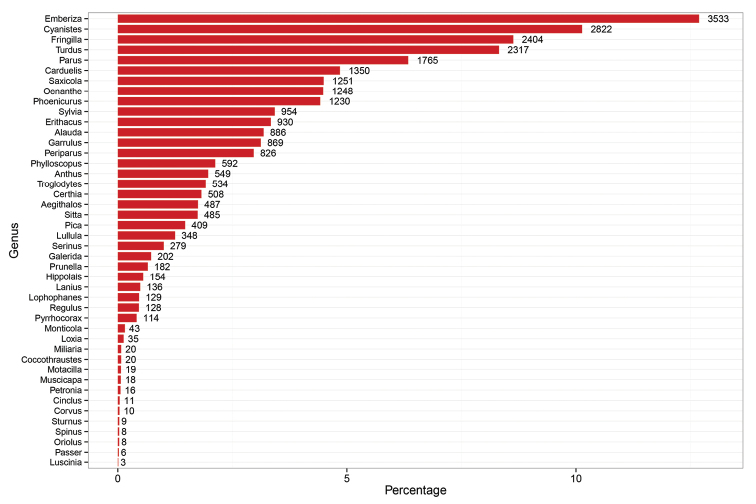
Distribution of records in the dataset according to genus.

This dataset includes a total of 27847 records of the order Passeriformes with 16 families represented (Figure 2). Nearly one third of the specimens belong to the family Fringillidae. A total of 44 genera are represented in this collection, with *Emberiza*, *Cyanistes*, *Turdus*, *Fringilla* and *Parus* having the highest number of records (Figure 3). Of this dataset 70 species appear in the European Red List (BirdLife International 2015): 67 are categorized as Least Concern, 2 is considered Near Threatened, and 1 is considered as Vulnerable (Table 1). According to the Spanish Red List (Madroño et al. 2004), 3 species in this dataset are placed in the Near Threatened category, 1 is listed as Vulnerable and 1 as Least Concern (Table 1).

**Table 1.** Conservation status of the species included in this dataset.v

Scientific name	European Red List ^a^	Spanish Red List ^b^	Birds Directive ^c^	Spanish Name ^d^	English Name ^e^
*Aegithalos caudatus* (Linnaeus, 1758)	LC	NE		Mito común	Long-tailed Tit
*Alauda arvensis* Linnaeus, 1758	LC	NE	IIB	Alondra común	Eurasian Skylark
*Anthus campestris* (Linnaeus, 1758)	LC	NE	I	Bisbita campestre	Tawny Pipit
*Anthus spinoletta* (Linnaeus, 1758)	LC	NE		Bisbita alpino	Water Pipit
*Carduelis cannabina* (Linnaeus, 1758)	LC	NE	II	Pardillo común	Common Linnet
*Carduelis carduelis* (Linnaeus, 1758)	LC	NE		Jilguero europeo	European Goldfinch
*Carduelis chloris* (Linnaeus, 1758)	LC	NE		Verderón común	European Greenfinch
*Carduelis spinus* (Linnaeus, 1758)	LC	NE		Jilguero lúgano	Eurasian Siskin
*Certhia brachydactyla* CL Brehm, 1820	LC	NE	I	Agateador europeo	Short-toed Treecreeper
*Cinclus cinclus* (Linnaeus, 1758)	LC	NE		Mirlo acuático europeo	White-throated Dipper
*Coccothraustes coccothraustes* (Linnaeus, 1758)	LC	NE		Picogordo común	Hawfinch
*Corvus corax* Linnaeus, 1758	LC	NE		Cuervo grande	Northern Raven
*Corvus monedula* Linnaeus, 1758	LC	NE	IIB	Grajilla occidental	Western Jackdaw
*Cyanistes caeruleus* (Linnaeus, 1758)	LC	NE		Herrerillo común	Eurasian Blue Tit
*Emberiza cia* Linnaeus, 1766	LC	NE		Escribano montesino	Rock Bunting
*Emberiza cirlus* Linnaeus, 1766	LC	NE		Escribano soteño	Cirl Bunting
*Emberiza hortulana* (Linnaeus, 1758)	LC	NE	I	Escribano hortelano	Ortolan Bunting
*Erithacus rubecula* Linnaeus, 1758	LC	NE		Petirrojo europeo	European Robin
*Fringilla coelebs* Linnaeus, 1758	LC	NE	I	Pinzón vulgar	Common Chaffinch
*Fringilla montifringilla* Linnaeus, 1758	LC	NE		Pinzón real	Brambling
*Galerida cristata* Linnaeus, 1758	LC	NE		Cogujada común	Crested Lark
*Galerida theklae* (CL Brehm,1858)	LC	NE	I	Cogujada montesina	Thekla Lark
*Garrulus glandarius* (Linnaeus, 1758)	LC	NE	IIB	Arrendajo euroasiático	Eurasian Jay
*Hippolais polyglotta* (Vieillot, 1817)	LC	NE		Zarcero políglota	Melodious Warbler
*Lanius meridionalis* Temminck, 1820	VU			Alcaudón norteño	Great Grey Shrike
*Lanius senator* Linnaeus, 1758	LC	NT		Alcaudón común	Woodchat Shrike
*Lophophanes cristatus* (Linnaeus, 1758)	LC			Herrerillo capuchino	European Crested Tit
*Loxia curvirostra* Linnaeus, 1758	LC	NE		Piquituerto común	Red Crossbill
*Lullula arborea* (Linnaeus, 1758)	LC	NE	I	Alondra Totovía	Woodlark
*Luscinia megarhynchos* CL Brehm, 1831	LC	NE		Ruiseñor común	Common Nightingale
*Miliaria calandra* (Linnaeus, 1758)	LC	NE		Escribano triguero	Corn Bunting
*Monticola saxatilis* (Linnaeus, 1766)	LC	NE		Roquero rojo	Common Rock Thrush
*Motacilla alba* Linnaeus, 1758	LC	NE		Lavandera blanca	White Wagtail
*Motacilla cinerea* Tunstall, 1771	LC	NE		Lavandera cascadeña	Grey Wagtail
*Muscicapa striata* (Pallas, 1764)	LC	NE		Papamoscas gris	Spotted Flycatcher
*Oenanthe hispanica* (Linnaeus, 1758)	LC	NT		Collalba rubia	Black-eared Wheatear
*Oenanthe oenanthe* (Linnaeus, 1758)	LC	NE		Collalba gris	Northern Wheatear
*Oriolus oriolus* (Linnaeus, 1758)	LC	NE		Oropéndola europea	Eurasian Golden Oriole
*Parus major* Linnaeus, 1758	LC	NE		Carbonero común	Great Tit
*Passer domesticus* (Linnaeus, 1758)	LC	NE		Gorrión común	House Sparrow
*Periparus ater* (Linnaeus, 1758)	LC	NE	I	Carbonero garrapinos	Coal Tit
*Petronia petronia* (Linnaeus, 1766)	LC	NE		Gorrión chillón	Rock Sparrow
*Phoenicurus ochruros* (SG Gmelin, 1774)	LC	NE		Colirrojo tizón	Black Redstart
*Phoenicurus phoenicurus* (Linnaeus, 1758)	LC	VU		Colirrojo real	Common Redstart
*Phylloscopus bonelli* (Vieillot, 1819)	LC	NE		Mosquitero papialbo	Western Bonelli’s Warbler
*Phylloscopus collybita* (Vieillot, 1817)	LC	NE		Mosquitero común	Common Chiffchaff
*Pica pica* (Linnaeus, 1758)	LC	NE	IIB	Urraca común	Eurasian Magpie
*Prunella collaris* (Scopoli, 1769)	LC	NE		Acentor alpino	Alpine Accentor
*Prunella modularis* (Linnaeus, 1758)	LC	NE		Acentor común	Dunnock
*Pyrrhocorax pyrrhocorax* (Linnaeus, 1758)	LC	NT	I	Chova piquirroja	Red-billed Chough
*Regulus ignicapillus* (Temminck, 1820)	LC	NE		Reyezuelo listado	Common Firecrest
*Saxicola rubetra* (Linnaeus, 1758)	LC	NE		Tarabilla norteña	Whinchat
*Saxicola rubicola* (Linnaeus, 1766)	LC	NE		Tarabilla común	African Stonechat
*Serinus citrinella* (Pallas, 1764)	LC	NE		Verderón serrano	Citril Finch
*Serinus serinus* (Linnaeus, 1766)	LC	NE		Serín Verdecillo	European Serin
*Sitta europaea* Linnaeus, 1758	LC	NE		Trepador azul	Eurasian Nuthatch
*Sturnus unicolor* Temminck, 1820	LC	NE		Estornino negro	Spotless Starling
*Sylvia atricapilla* (Linnaeus, 1758)	LC	NE		Curruca capirotada	Eurasian Blackcap
*Sylvia cantillans* (Pallas, 1764)	LC	NE		Curruca carrasqueña	Subalpine Warbler
*Sylvia communis* Latham, 1787	LC	NE		Curruca zarcera	Common Whitethroat
*Sylvia conspicillata* Temminck, 1820	LC	LC		Curruca tomillera	Spectacled Warbler
*Sylvia melanocephala* (Gmelin, 1789)	LC	NE		Curruca cabecinegra	Sardinian Warbler
*Sylvia undata* (Boddaert, 1783)	NT	NE	I	Curruca rabilarga	Dartford Warbler
*Troglodytes troglodytes* (Linnaeus, 1758)	LC	NE	I	Chochín común	Eurasian Wren
*Turdus iliacus* Linnaeus, 1758	NT	NE	IIB	Zorzal alirrojo	Redwing
*Turdus merula* Linnaeus, 1758	LC	NE	IIB	Mirlo común	Common Blackbird
*Turdus philomelos* CL Brehm, 1831	LC	NE	IIB	Zorzal común	Song Thrush
*Turdus pilaris* Linnaeus, 1758	LC	NE	IIB	Zorzal real	Fieldfare
*Turdus torquatus* Linnaeus, 1758	LC	NE		Mirlo capiblanco	Ring Ouzel
*Turdus viscivorus* Linnaeus, 1758	LC	NE	IIB	Zorzal charlo	Mistle Thrush

a European Red List of Birds (BirdLife International 2015).

b Red Book of the birds of Spain (Madroño et al. 2004).

c Species included in the Birds Directive Annexes (EC 1979)

d Spanish common names (Gutiérrez et al. 2012, De Juana et al. 2004, 2005, 2007, 2009a, 2009b, 2010a, 2010b).

e English common names (Gill and Donsker 2015).

LC
: Least Concern ; NE : Not Evaluated ; NT : Near Threatened ; VU : Vulnerable .

## Taxonomic ranks

Kingdom: Animalia

Phylum: Chordata

Subphylum: Craniata

Class: Aves

Order: Passeriformes

Family: Aegithalidae, Alaudidae, Certhiidae, Cinclidae, Corvidae, Fringillidae, Laniidae, Motacillidae, Muscicapidae, Paridae, Passeridae, Phylloscopidae, Sittidae, Sturnidae, Sylviidae, Turdidae

Genus: *Aegithalos*, *Alauda*, *Anthus*, *Carduelis*, *Certhia*, *Cinclus*, *Coccothraustes*, *Corvus*, *Cyanistes*, *Emberiza*, *Erithacus*, *Fringilla*, *Galerida*, *Garrulus*, *Hippolais*, *Lanius*, *Lophophanes*, *Loxia*, *Lullula*, *Luscinia*, *Miliaria*, *Monticola*, *Motacilla*, *Muscicapa*, *Oenanthe*, *Oriolus*, *Parus*, *Passer*, *Periparus*, *Petronia*, *Phoenicurus*, *Phylloscopus*, *Pica*, *Prunella*, *Pyrrhocorax*, *Regulus*, *Saxicola*, *Serinus*, *Sitta*, *Spinus*, *Sturnus*, *Sylvia*, *Troglodytes*, *Turdus*

## Spatial coverage

**Bounding box for covered area**: 36°52’12”N and 37°15’36”N Latitude; 3°41’24”W and 2°33’36”W Longitude

**Figure 4.**
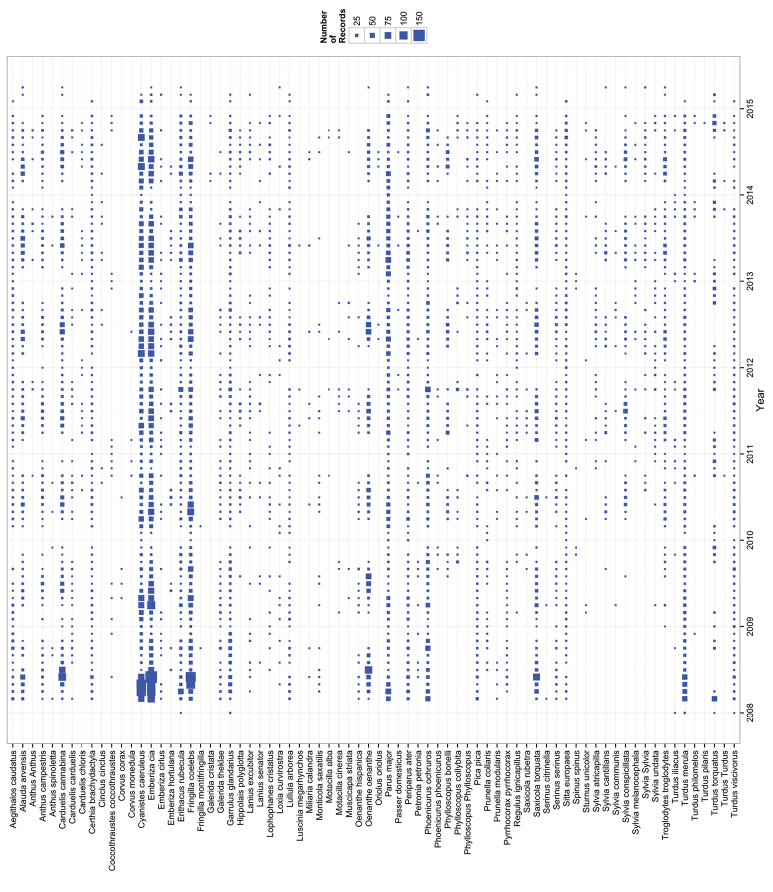
Temporal coverage of the dataset. For each taxon (y-axis) the temporal coverage is shown including a point. Point size is proportional to monthly records of each taxon.

**Temporal coverage**: Observations in the collection included in this data paper date from March 2008 to April 2015 (Figure 4).

**Collection name**: Dataset of Passerine bird communities in a Mediterranean high mountain (Sierra Nevada, Spain)

**Collection identifier**: http://www.gbif.es/ipt/resourcve?r=passerine

## Methods

**Study extent description**: This dataset covers four representative habitats within the Sierra Nevada mountain range: Pyrenean oak forest, thorny thickets on the edge of the forest, common juniper and Spanish juniper scrublands, and high-summit ecosystems. These ecosystems were selected based on criteria of singularity and ecological functionality in the context of Sierra Nevada (Barea-Azcón et al. 2012, 2014) and can be described as follows:

Pyrenean oak forest: Mediterranean woodland composed mainly of relict *Quercus pyrenaica* and some dominant scrubland species (i.e. *Berberis hispanica*, *Prunus ramburii*, *Rosa canina*, *Crataegus monogyna and Adenocarpus decorticans*). These forests show strong evidence of past management that has determined their current structure and diversity. This management is based on mainly charcoal production, pastureland creation, and wood harvesting until the 1950s, so that the current trees are mostly resprouts of individuals 60 to 70 years old. The target localities (*n*=*4*) are located at an average elevation of 1650 m a.s.l. (1600-1750 m a.s.l.) and are distributed in the southern, western, northern, and eastern slopes of Sierra Nevada, reflecting all the ecological conditions of the Pyrenean oak forests in the study area (Pérez-Luque et al. 2013).

Thorny scrubs: Typical areas dominated by thorny thickets on the edge of the forest or as result of recent colonization of abandoned arable lands. *Berberis hispanica*, *Prunus ramburii*, *Rosa canina*, *Crataegus monogyna* are dominant but accompanied by other species such as *Lonicera arborea* or even *Sorbus* spp. This open habitat is very important for breeding birds in the study area and also for winter-visiting species due to a great production of fruits from the end of the summer to the beginning of winter. Transects (*n*=*4*) in this habitat are located between 1450 and 2060 m a.s.l. (average: 1790 m a.s.l.).

Common juniper and Spanish juniper scrublands: vegetation in these localities is composed mainly of common juniper (*Juniperus communis*), Spanish juniper (*Juniperus sabina*). *Cytisus galianoi* and *Genista baetica* are also important species in these ecosystems. These scrublands rarely exceed 60 cm in height and appear intermingled with rocks and stony ground. Transects (*n*=*4*) located in this ecosystems cover an elevational range from 2000 to 2300 m a.s.l. (average: 2150 m a.s.l.).

High-summit ecosystems: composed by typical Alpine landscape. These ecosystems are characterized by rocky outcrops that originated from glacial activity, pastureland, small snow beds, and glacial lagoons. The four transects representing this Mediterranean high-mountain habitat span an elevational gradient from 2280 to 3100 m a.s.l., with an average elevation of 2580 m a.s.l.

**Sampling description**: The sampling procedure was the line-transect method (Verner 1985), with a bandwidth of 100 m, with 50 m on each side of the line (Barea-Azcón et al. 2014). Each 50 m band was divided into five ranges parallel to the line transect (comprising a 10 m width each one). A total of 16 transects were sampled with lengths of 1.9 to 3 km (Table 2). Sight and sound records within the sample area were considered contacts. All transects were sampled in the early morning, under appropriate climatic conditions. The observer walked at a constant speed of 2 to 4 km/h. Transects are repeated at least once per month, snow cover permitting. This implies that the sites located at the higher elevations were sampled only from late spring to early autumn.

**Table 2.** Information about transects sampled to collect data included in this dataset.

Transect name	Length (m)	Habitat type	Longitude	Latitude	Province	Municipality	Elevation (m asl)
Robledal de Cáñar	2556	Pyrenean oak Forest	-3.4292	36.9532	Granada	Cáñar	1736
Robledal de Dílar	2553		-3.4779	37.0582	Granada	Dílar	1605
Cortijo del Hornillo	3044		-3.3680	37.1246	Granada	Güejar Sierra	1585
Dehesa del Camarate	2805		-3.2537	37.1797	Granada	Lugros	1575
Dehesa del Río Dúrcal	3292	Thorny thickets	-3.4825	37.0255	Granada	Dúrcal	2033
Collado de Matas Verdes	2237		-3.4470	37.0909	Granada	Monachil	1918
El Purche	1944		-3.4780	37.1311	Granada	Monachil	1453
Lanteira	2515		-3.1725	37.1409	Granada	Lanteira	1794
Collado del Sabinar	2745	Juniper scrublands	-3.4184	37.1199	Granada	Güejar Sierra	2036
Campos de Otero	2264		-3.3930	37.1100	Granada	Güejar Sierra	2143
Loma Papeles	2539		-3.3401	37.1434	Granada	Güejar Sierra	2113
Dehesa de las Hoyas	2436		-3.3173	37.1724	Granada	Güejar Sierra	2074
Laguna Seca	2530	High-summit ecosystems	-2.9615	37.0992	Granada	Huéneja	2295
Aguas Verdes	2431		-3.3589	37.0540	Granada	Capileira	3149
Hoya Mora	2046		-3.3771	37.0896	Granada	Güejar Sierra	2407
Papeles alto	2309		-3.3098	37.1357	Granada	Güejar Sierra	2420

**Method step description**: All data were stored in a normalized database (PostgreSQL) and incorporated into the Information System of Sierra Nevada Global-Change Observatory. Taxonomic and spatial validations were made on this database (see *Quality-control description*). A custom-made SQL view of the database was performed to gather occurrence data and other variables associated with occurrence data, specifically:

Bird Count: number of individuals recorded by the observer within transect (see *Sampling description*)

Distance: distance of the contact (bird) from transect line. The distance was estimated by eye.

The occurrence and measurement data were accommodated to fulfil the Darwin Core Standard (Wieczorek et al. 2009, 2012). We used Darwin Core Archive Validator tool (http://tools.gbif.org/dwca-validator/) to check whether the dataset met Darwin Core specifications. The Integrated Publishing Toolkit (IPT v2.0.5) (Robertson et al. 2014) of the Spanish node of the Global Biodiversity Information Facility (GBIF) (http://www.gbif.es/ipt) was used both to upload the Darwin Core Archive and to fill out the metadata.

The Darwin Core elements for the occurrence data included in the dataset were: occurrenceId, modified, language, basisOfRecord, institutionCode, collectionCode, catalogNumber, scientificName, kingdom, phylum, class, order, family, genus, specificEpithet, scientificNameAuthorship, continent, country, countryCode, stateProvince, county, locality, minimumElevationInMeters, maximumElevationInMeters, decimalLongitude, decimalLatitude, coordinateUncertaintyinMeters, geodeticDatum, recordedBy, day, month, year, EventDate.

For the measurement data, the Darwin Core elements included were: occurrenceId, measurementID, measurementType, measurementValue, measurementAccuracy, measurementUnit, measurementDeterminedDate, measurementDeterminedBy, measurementMethod.

**Quality control description**: The sampling transects were georeferenced using a hand held GPS device (WGS 84 Datum) with an accuracy of ±5 m. We also used colour digital orthophotographs provided by the Andalusian Cartography Institute and GIS (ArcGIS 9.2; ESRI, Redlands, California, USA) to verify that the geographical coordinates of the transects were correct (Chapman and Wieczorek 2006).

For field identification, several field guides were used (De Juana and Varela 2000, Jonsson 2001). The scientific names were checked with database of the IOC World Bird List (v 5.52) (Gill and Donsker 2015). We also used the R package taxize (Chamberlain and Szocs 2013, Chamberlain et al. 2014) to verify the taxonomical classification.

In addition, we performed validation procedures (Chapman 2005a, 2005b) (geopraphic coordinate format, coordinates within country/provincial boundaries, absence of ASCII anomalous characters in the dataset) with DARWIN_TEST (v3.2) software (Ortega-Maqueda and Pando 2008).

## Dataset description

**Object name**: Darwin Core Archive Dataset of Passerine bird communities in a Mediterranean high mountain (Sierra Nevada, Spain)

**Character encoding**: UTF-8

**Format name**: Darwin Core Archive format

**Format version**: 1.0

**Distribution**: http://www.gbif.es/ipt/resource?r=passerine

**Publication date of data**: 2015-10-08

**Language**: English

**Licenses of use**: This “*Dataset of Passerine bird communities in a Mediterranean high mountain (Sierra Nevada, Spain)*” is licensed under and made available under the Creative Commons Attribution Non Commercial (CC-BY-NC) 4.0 License http://creativecommons.org/licenses/by-nc/4.0/legalcode

**Metadata language**: English

**Date of metadata creation**: 2015-10-08

**Hierarchy level**: Dataset
